# ﻿A new species and a new record of *Phlomoides* (Lamiaceae) from Xizang, China

**DOI:** 10.3897/phytokeys.246.129057

**Published:** 2024-08-27

**Authors:** Yue Zhao, Ya-Ping Chen, Rui-Zhu Bai, Colin A. Pendry, Alexander P. Sukhorukov, Chun-Lei Xiang

**Affiliations:** 1 State Key Laboratory of Desert and Oasis Ecology, Xinjiang Institute of Ecology and Geography, Chinese Academy of Sciences, Urumqi 830011, China Xinjiang Institute of Ecology and Geography, Chinese Academy of Sciences Urumqi China; 2 Key Laboratory of Phytochemistry and Natural Medicines, Kunming Institute of Botany, Chinese Academy of Sciences, Kunming 650201, China Kunming Institute of Botany, Chinese Academy of Sciences Kunming China; 3 Royal Botanic Garden Edinburgh, Edinburgh EH35LR, UK Royal Botanic Garden Edinburgh Edinburgh United Kingdom; 4 Department of Higher Plants, Biological Faculty, Lomonosov Moscow State University, Moscow 119234, Russia Lomonosov Moscow State University Moscow Russia

**Keywords:** Lamioideae, Phlomideae, *Phlomis*, taxonomy, Tibet

## Abstract

*Phlomoidesbomiensis*, a new species in Bomi County, Xizang, China, was described and illustrated. In addition, *Phlomoideslongidentata*, previously only known from Nepal and Bhutan, is newly recorded from Dingri County, Xizang, China. The phylogenetic placement of both species within the genus was analysed using nine plastid DNA markers (*atpB-rbcL*, *psbA-trnH*, *rpl16*, *rpl32-trnL*, *rps16*, *trnK*, *trnL-trnF*, *trnS-trnG*, *trnT-trnL*). Both species have brown-black trichomes inside the upper corolla lip and nested within the same subclade of Clade II. A diagnostic key to the *Phlomoides* species belonging to this subclade is provided.

## ﻿Introduction

As revealed by phylogenetic studies, the resurrected genus *Phlomoides* Moench includes traditionally defined PhlomisL.sect.Phlomoides (Moench) Briq., *Eremostachys* Ledeb., *Lamiophlomis* Kudô, *Metastachydium* Airy Shaw ex C.Y.Wu & H.W.Li, *Notochaete* Benth., *Pseudomarrubium* Popov, *Paraeremostachys* Adylov, Kamelin & Makhm. and *Pseuderemostachys* Popov (Scheen et al. 2010; Bendiksby et al. 2011; Mathiesen et al. 2011; Salmaki et al. 2012; Zhao et al. 2023a, b). With this updated circumscription, *Phlomoides* encompasses approximately 180 species. The genus is characterised by perennial habits, often tuberous roots, cordate to triangular-ovate, entire to bipinnatisect leaves, 5-toothed calyx, dome-shaped and apically hairy upper corolla lips. Notably, some species of *Phlomoides* have been used in traditional medicine, such as *P.rotata* (Benth. ex Hook.f.) Mathiesen, *P.betonicoides* (Diels) Kamelin & Makhm. and *P.medicinalis* (Diels) Kamelin & Makhm. (Peng and Xiang 2017).

China is one of the three diversity centres of *Phlomoides*, boasting 60 species and 17 varieties, of which 39 species are distributed in the Tibetan Plateau, Himalaya and Hengduan Mountains (Wu and Li 1977; Li and Hedge 1994; Zhao et al. 2021a, b, 2022, 2023a, 2024a, b, c). However, the diversity of *Phlomoides* in these areas remains understudied. Recent discoveries have shed light on this hidden species diversity, with the description of four species new to science within these regions. Amongst them, *Phlomoidesliangwangshanensis* Y.Zhao, H.L.Zheng & C.L.Xiang and *P.henryi* Y.Zhao & C.L.Xiang are distributed in the Hengduan Mountains and *P.cuonaensis* Y.Zhao, C.L.Xiang & Sukhor. and *P.longidentata* Pendry are found in the Himalayas (Pendry 2021; Zhao et al. 2021a, 2024a, b).

During our field trip in Xizang, we found one new species of *Phlomoides* in Bomi County and one new record of the genus in Dingri County. Both species have brown-black trichomes inside the upper corolla lip. In this study, we provide detailed descriptions and illustrations of both the new and newly-recorded species. In addition, we make detailed morphological comparisons with other species which have brown-black trichomes in the upper corolla lip. This comparative analysis aims to facilitate accurate identification and classification within the genus *Phlomoides*.

## ﻿Material and methods

### ﻿Taxon sampling

Taxon sampling is primarily based on our previous molecular phylogenetic study (Zhao et al. 2024a). For this particular study, the putative new species (*Phlomoidesbomiensis* C.L.Xiang & Y.Zhao), the newly-recorded species (*Phlomoideslongidentata*), as well as three morphologically related species [three individuals of *Phlomoidestibetica* (C.Marquand & Airy Shaw) Kamelin & Makhm., one individual each of *Phlomoidesrotata* (Benth. ex Hook.f.) Mathiesen and *Phlomoidesnana* (C.Y.Wu) Y.Zhao & C.L.Xiang] were sequenced for the first time. In total, our molecular phylogenetic analyses comprised 59 individuals, representing 53 Chinese species of *Phlomoides*. In addition, three species of *Phlomis* were selected as outgroup.

### ﻿DNA extraction, selection of markers and molecular phylogenetic analyses

The CTAB method was used to extract total genomic DNA from silica gel dried leaf materials (Doyle and Doyle 1987). Sequences of nine plastid DNA markers (*atpB-rbcL*, *psbA-trnH*, *rpl16*, *rpl32-trnL*, *rps16*, *trnK*, *trnL-trnF*, *trnS-trnG*, *trnT-trnL*) were chosen for the phylogenetic reconstruction. Primers, polymerase chain reaction (PCR), sequencing and alignment were carried out according to procedures used in Zhao et al. (2024a). The sequences, newly generated in this study together with their GenBank accession numbers, are listed in Suppl. material 1.

Bayesian Inference (BI) and Maximum Likelihood (ML) were used for phylogenetic reconstruction. Detailed settings for BI and ML analyses followed Zhao et al. (2024a). TreeGraph2 (Stöver and Müller 2010) was applied to visualise and edit all trees.

### ﻿Morphological and taxonomy study

We thoroughly examined herbarium specimens or their digital images from the following Herbaria: B, BM, C, CDBI, E, FI, GH, HIB, IBSC, K, KUN, KYO, L, LE, M, MA, MAO, MO, MW, NAS, P, PE and TI. During our field investigations, we observed and documented important diagnostic characteristics of *Phlomoides* species. These observations were complemented by high-resolution photographs taken in their natural habitats. Trichome morphology was observed and measured under a Leica DM2500 optical microscope (Leica Microsystems GmbH, Wetzlar, Germany).

## ﻿Results and discussion

### ﻿Sequence characterisation

In total, 558 sequences were included for phylogenetic analyses, of which 63 sequences were newly sequenced in this study and they were submitted to GenBank (Suppl. material 1). The aligned cpDNA dataset was 9,222 nucleotides in length (2,382 bp for *atpB-rbcL*, 439 bp for *psbA-trnH*, 1,365 bp for *rpl16*, 677 bp for *rpl32-trnL*, 968 bp for *rps16*, 954 bp for *trnK*, 869 bp for *trnL-trnF*, 825 bp for *trnS-trnG* and 743 bp for *trnT-L*, respectively), of which 883 bp (9.57%) are variable. Characteristics for all datasets are listed in Table 1.

**Table 1.** The statistics of all datasets for phylogenetic analysis.

Datasets	No. Taxa	Nucleotides (with ambiguous sites excluded) [bp]	GC content (%)	No. constant sites [bp]	No. variable sites [bp]	No. parsimony- informative sites [bp]
*atpB-rbcL*	62	2382	39.3	2244	138	88
*psbA-trnH*	62	439	32.7	384	55	29
*rpl16*	62	1365	36.2	1211	154	91
*rpl32-trnL*	62	677	31.7	576	101	66
*rps16*	62	968	35.6	891	77	45
*trnK*	62	954	34	844	110	65
*trnL-trnF*	62	869	36.1	794	75	42
*trnS-trnG*	62	825	33.5	738	87	50
*trnT-trnL*	62	743	29.4	657	86	56
combined	62	9222	35.5	8339	883	532

### ﻿Phylogenetic reconstruction

**Figure 1.**
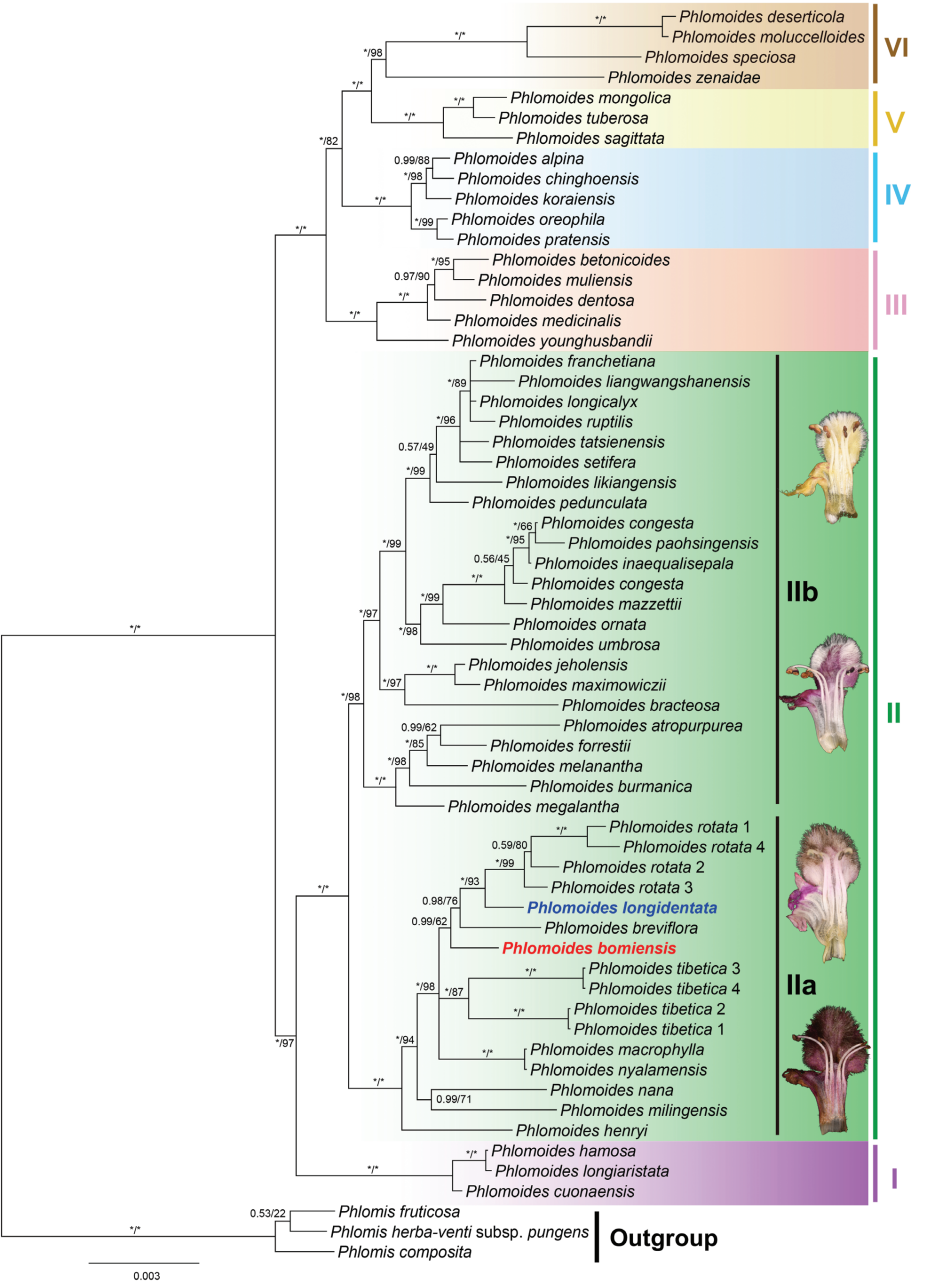
Phylogeny of *Phlomoides* inferred by Bayesian Inference (BI), based on the combined plastid dataset cpDNA. Support values displayed on the branches follow the order BI-PP/ML-BS (“ * ” indicates PP = 1.00 or BS = 100%).

The phylogenetic analyses using both Bayesian Inference (BI) and Maximum Likelihood (ML) methods yielded largely congruent tree topologies. Therefore, only the Bayesian 50% majority rule consensus tree is presented, with posterior probabilities (PP) and bootstrap values (BS) indicated near nodes (Fig. 1).

Consistent with our previous molecular phylogenetic analyses (Zhao et al. 2024a), six well-supported clades can be recognised (Fig. 1). Clade II includes the majority of species characterised by having linear-tuberous roots, without persistent basal leaves and glabrous nutlets (Zhao et al. 2024a). In the present study, we found that Clade II can be divided into two major subclades with strong support values. Subclade IIa (Fig. 1: PP = 1.00/BS = 100%) contains the putative new species (*P.bomiensis*), as well as *P.longidentata*. Most species in this subclade are characterised by brown-black trichomes inside the upper corolla lip, except for *P.rotata* and *P.henryi* (Zhao et al. 2024c). This subclade unites ten species [*P.rotata*, *P.longidentata*, *P.breviflora* (Benth.) Kamelin & Makhm., *P.bomiensis*, *P.tibetica*, *P.macrophylla* (Benth.) Kamelin & Makhm., *P.nyalamensis* (H.W.Li) Y.Zhao & C.L.Xiang, *P.nana*, *P.milingensis* (C.Y.Wu & H.W.Li) Kamelin & Makhm. and *P.henryi*]. All the species in subclade IIb (Fig. 1: 1.00/98%) have white and transparent trichomes inside the upper corolla lip.

*Phlomoideslongidentata* is sister to *P.rotata* which is represented by four individuals (Fig. 1: 1.00/93%) and these species are sisters to *P.breviflora* (Fig. 1: 0.98/76%). *P.bomiensis* is sister to all three species (Fig. 1: 0.99/62%).

### ﻿Morphological comparison

The species within subclade IIa are distributed in Tibetan Plateau, Himalaya and Hengduan Mountains. Here, we provide a morphological comparison of the ten species grouped in the subclade IIa (Table 2) to evaluate the most significant diagnostic traits. The trichome colour, floral leaves and calyx teeth were identified as having taxonomic significance within this subclade. A key is provided to differentiate these species.

**Table 2.** Comparative morphological characters amongst *Phlomoidesbomiensis*, *P.longidentata* and their related species.

	Height	Basal leaves	Floral leaf petiole length	Flower colour	Trichome colour of upper corolla lip	Apical part of calyx tube
*P.rotata*	2.5–10 cm	+	Lack obvious petiole	Purple or white	White	Broadly triangular
*P.longidentata*	50–100 cm	–	5–100 mm	Light purple to pink	Black or brown	Emarginate
*P.breviflora*	60–150 cm	–	30–130 mm	Purple	Black or brown	Subtruncate or broadly triangular
*P.bomiensis*	50–180 cm	–	5–70 mm	Purple	Black or brown	Subtruncate or slightly emarginate
*P.tibetica*	10–30 (50) cm	+	0 (5) mm	Purple to light purple	Black or brown	Subtruncate or slightly emarginate
*P.macrophylla*	100–200 cm	–	1–10 (50) mm	White to light pink	Black or brown	Emarginate
*P.nyalamensis*	100–200 cm	–	1–10 (50) mm	Purple	Black or brown	Emarginate
*P.nana*	30–50 cm	+	2–5 (10) mm	White to light pink	Black or brown	Emarginate
*P.milingensis*	15–50 cm	+	2–10 mm	Purple	Black or brown	Subtruncate or slightly emarginate
*P.henryi*	100–150 cm	–	5–35 mm	Light purple to pink	White	Truncate

## ﻿Taxonomic treatment

### ﻿ Phlomoides bomiensis

Taxon classificationPlantaeLamialesLamiaceae

C.L.Xiang & Y.Zhao sp. nov.

AD753262-A8BC-5A31-8B24-EC105F8EF465

urn:lsid:ipni.org:names:77347473-1

Fig. 2

#### Type.

China, Xizang (Tibet), Linzhi City, Bomi County, on the road from Bomi to Motuo, near Galongla Tunnel, 29°48′22.4″N, 95°42′2.45″E, alt. 3454 m, 22 Aug 2023, *Y. Zhao, R.Z. Bai, Q. Tian & M.L. Qian XCL2584* (holotype: KUN 1614346!; isotypes: KUN 1614347!, KUN 1614348!, KUN 1614349!).

#### Diagnosis.

*Phlomoidesbomiensis* is morphologically most similar to *P.nyalamensis* and *P.breviflora*. These species are often taller than 1 m and have a purple corolla with brown to black trichomes inside the upper lip. It differs from *P.nyalamensis* by its subtruncate to slightly emarginate calyx teeth, posterior filaments with reflexed appendages at base and floral leaves with obvious petioles (vs. obviously emarginate calyx teeth, posterior filaments without appendages and sessile upper floral leaves). It differs from *P.breviflora* by its corolla that is longer than 2 cm and its oblong nutlets (vs. corolla often shorter than 1.5 cm and inflated globose nutlets). The differences between the ten species from subclade IIa are listed in Table 2.

#### Description.

Perennial herbs. ***Roots*** robust, linear-tuberous. ***Stems*** 0.5–1.8 m tall, subquadrangular, robust, lower stem part glabrous, upper part with simple trichomes. ***Basal leaves*** absent; ***stem leaves*** with petioles 5–14 cm long, with simple trichomes, blade cordate, papery, 8–21 × 8–18 cm, adaxially green with simple trichomes, denser on vein, abaxially light green, only with sparse simple trichomes on vein, margin crenate, apex acute. ***Verticillasters*** axillary, 8–18-flowered; ***floral leaves*** with petioles 0.5–7 cm long, blade cordate to truncate, 3–15 × 2–12 cm, gradually reduced upwards; ***bracts*** linear to lanceolate, 8–10 mm long, with sparse long (2–3 mm) simple trichomes. ***Calyx*** tubular, 12 × 5 mm, conspicuously 10-veined with sparse simple (2–3 mm long) trichomes on veins, tube subtruncate to slightly emarginate, teeth 5, ca. 1 mm long, apical spines 1 mm long. ***Corolla*** purple, 21–23 mm long, 2-lipped; posterior lip ca. 8–10 mm long, galeate, densely stellate tomentose outside, margin denticulate, brown to black bearded inside; anterior lip 3-lobed, ca. 7 × 8 mm, middle lobe largest, oblong, ca. 5 × 3 mm, lateral lobes ovate; tube glabrous outside, ca. 11 mm, annulate pilose inside. ***Stamens*** 4, included within posterior lip, with “cobweb-like” indumentum, posterior filaments with reflexed appendages at base. ***Style*** unequally 2-lobed. Nutlets oblong, apex truncate, glabrous.

**Figure 2.**
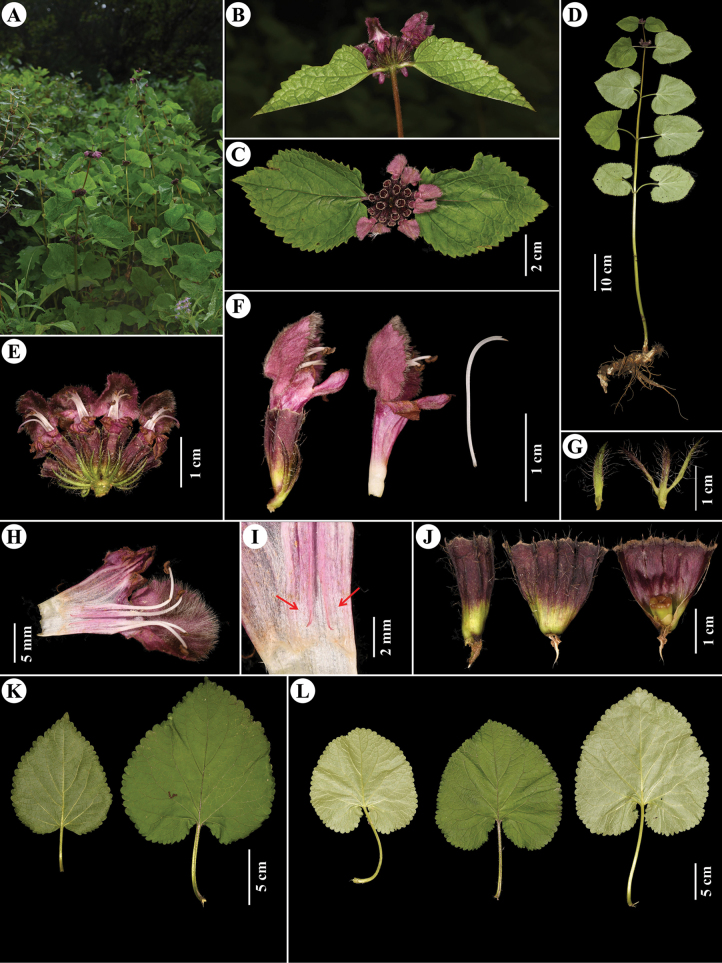
*Phlomoidesbomiensis* C.L.Xiang & Y.Zhao **A** habitat **B, C** verticillaster with floral leaves **D** plant **E** verticillaster **F** flower **G** bracts **H** dissected flower **I** appendages at base of posterior filaments (arrow) **J** calyx and dissected calyx **K** floral leaves **L** Stem leaves (Photographed by Yue Zhao).

#### Phenology.

Flowering from August to September, fruiting from October to November.

#### Distribution and habitat.

**Figure 3.**
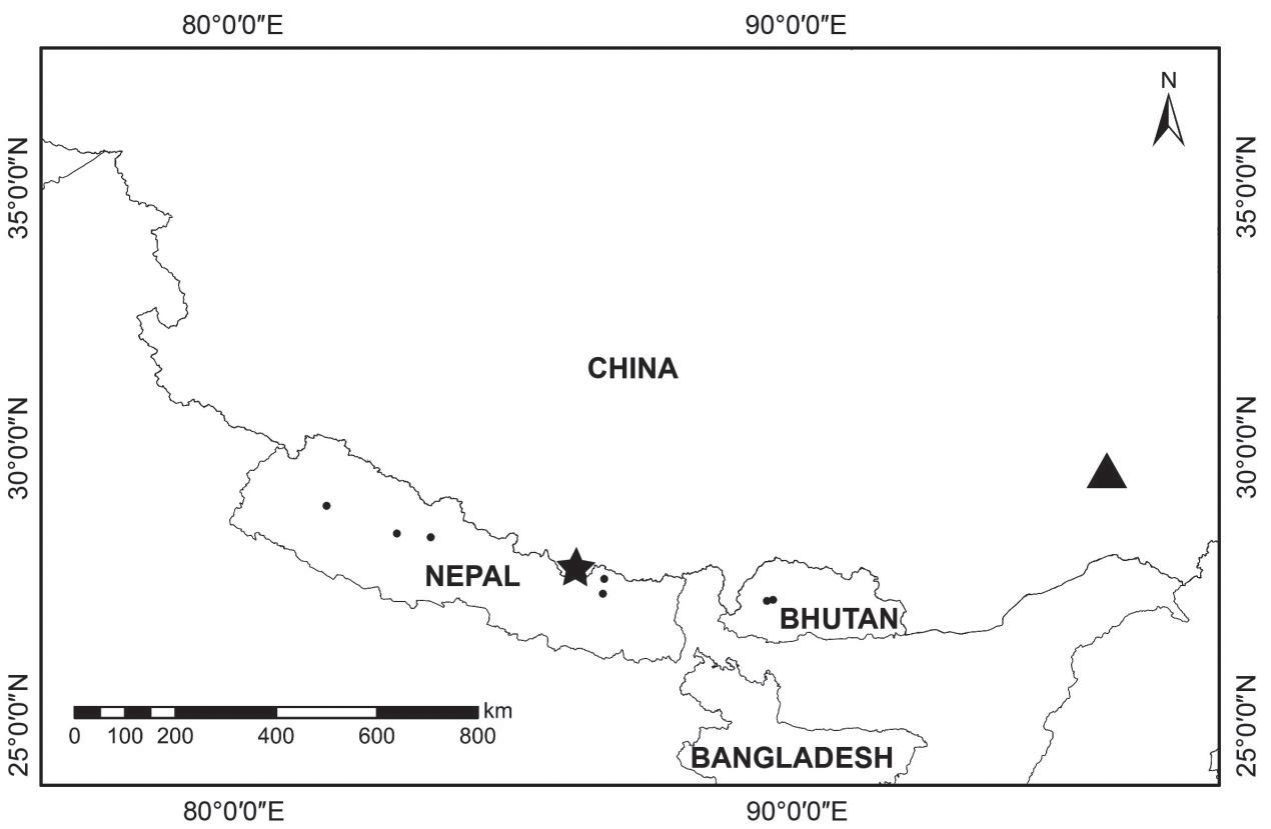
Distribution of *Phlomoidesbomiensis* (black triangle), new location of *P.longidentata* (black star) and known localities of *P.longidentata* (black circle).

Based on our field collections and previously collected specimens, *P.bomiensis* is known to occur in Bomi County and Motuo County, Xizang (Tibet), China. It grows in forests and forest margins at altitudes between 3400 and 4200 m (Fig. 3).

#### Etymology.

The specific epithet refers to the name of the Bomi County in Xizang Autonomous Region, where the new species was discovered.

#### Chinese name (assigned here).

bō mì cǎo cāo sū (波密草糙苏)

#### Additional specimen examined (paratypes).

China. Xizang (Tibet): • Linzhi City, Motuo County, Northern Galongla Pass, alt. 3500–3700 m, 20 Aug 1982, *S.Z. Cheng & B.S. Li 000315* (PE 00923558!, PE 00832483!, PE 00832484!) • Linzhi City, Bomi County, 30 km away from Zhamo Road, alt. 4200 m, 27 Jul 2010, *South Tibet Expedition Team STET1237* (PE 02328210!) • Linzhi City, Bomi County, Galongla Mountain, alt. 3879 m, 17 Jul 2022, *J.F. Xiao, H.Z. Feng & Er.F. Huang XJF114* (KUN 1614350!).

*Phlomoidesbomiensis* was first collected more than 40 years ago (*S.Z. Cheng & B.S. Li 00315*; PE 00923558!, PE 00832483!, PE 00832484!), but the specimens were then identified as Phlomoidesumbrosa(Turcz.)Kamelin & Makhm.var.australis (Hemsl.) C.L.Xiang & H.Peng. However, Phlomoidesumbrosavar.australis was distinguished from *P.bomiensis* by having white or transparent trichomes on upper corolla and subsessile floral leaves (vs. brown-black trichomes on upper corolla and floral leaves with petioles 0.5–7 cm long). Another specimen of *P.bomiensis* was collected in 2010 (*South Tibet Expedition STET1237*; PE 02328210!), but it was misidentified as *Phlomoidestibetica*. The differences between *Phlomoidestibetica* and *P.bomiensis* are provided in Table 2.

### ﻿ Phlomoides longidentata

Taxon classificationPlantaeLamialesLamiaceae

Pendry, Edinburgh J. Bot. 78: 4, 2021.

A3779EE5-1943-5556-A645-4CE6434D3775

Fig. 4

#### Type.

Nepal, Solukhumbu District, Namche Bazar, above bridge over Dudh Kosi. 27°47′31′′N, 86°42′57′′E, alt. 3060 m, 12 Sep 2006, *DNEP3 BX36* (holotype: E!; isotypes: KATH!, TI!, TUCH!).

#### Diagnosis.

*Phlomoideslongidentata* is a recently described species from Bhutan and Nepal (Pendry 2021). Specimens of *P.longidentata* were collected by Chinese collectors as early as 1959 (*Anonymous 752*; PE 00832482!), when it was identified as *P.umbrosa* and P.umbrosavar.australis. During our field investigations in Rongxia Town, we rediscovered this species in the wild. It rather looks like a perennial herb, but not annual as described by Pendry (2021). Here we provide description of this species.

#### Description.

Perennial herbs. ***Roots*** delicate, thin, linear-tuberous. ***Stems*** 30–60 cm tall, subquadrangular, unbranched, stellate pilose. ***Basal leaves*** absent; ***stem leaves*** with petioles 4–19 cm long, with stellate (with a long central ray) and simple trichomes, blade cordate, papery, 4–14 × 4–15 cm, adaxially green with sparse simple trichomes, denser and longer on the main vein, abaxially light green, with dense stellate trichomes with equal rays, (stellate trichomes denser and with longer central ray on the veins), base cordate, margin serrate or crenate, apex acute. ***Verticillasters*** axillary, 8–20-flowered; ***floral leaves*** with petioles 0.5–10 cm long, blade lanceolate, base cordate to truncate, 4.5–13 × 2–11 cm, gradually reduced upwards; ***bracts*** subulate, 7–8 mm long, with sparse long simple trichomes, ca. 2 mm long. ***Calyx*** tubular, 10–11 × 4–5 mm, pubescent outside with sparse stellate trichomes with equal rays outside (10 tubular veins have longer central rays); tube apically emarginate; teeth 5, unequal, two longer teeth 4 mm long, three shorter teeth 2–3 mm long. ***Corolla*** light purple to pink, 19–22 mm long, 2-lipped; posterior lip 7–8 mm long, galeate, densely stellate tomentose outside, margin denticulate, brown to black-bearded inside; anterior lip 3-lobed, ca. 6 × 8 mm, middle lobe largest, oblong, ca. 5 × 3 mm, lateral lobes ovate; tube glabrous outside, ca. 12 mm, annulate pilose inside. ***Stamens*** 4, included within posterior lip, with “cobweb-like” indumentum, posterior filaments with reflexed appendages at base. ***Style*** unequally 2-lobed. ***Nutlets*** oblong, apex truncate, glabrous.

**Figure 4.**
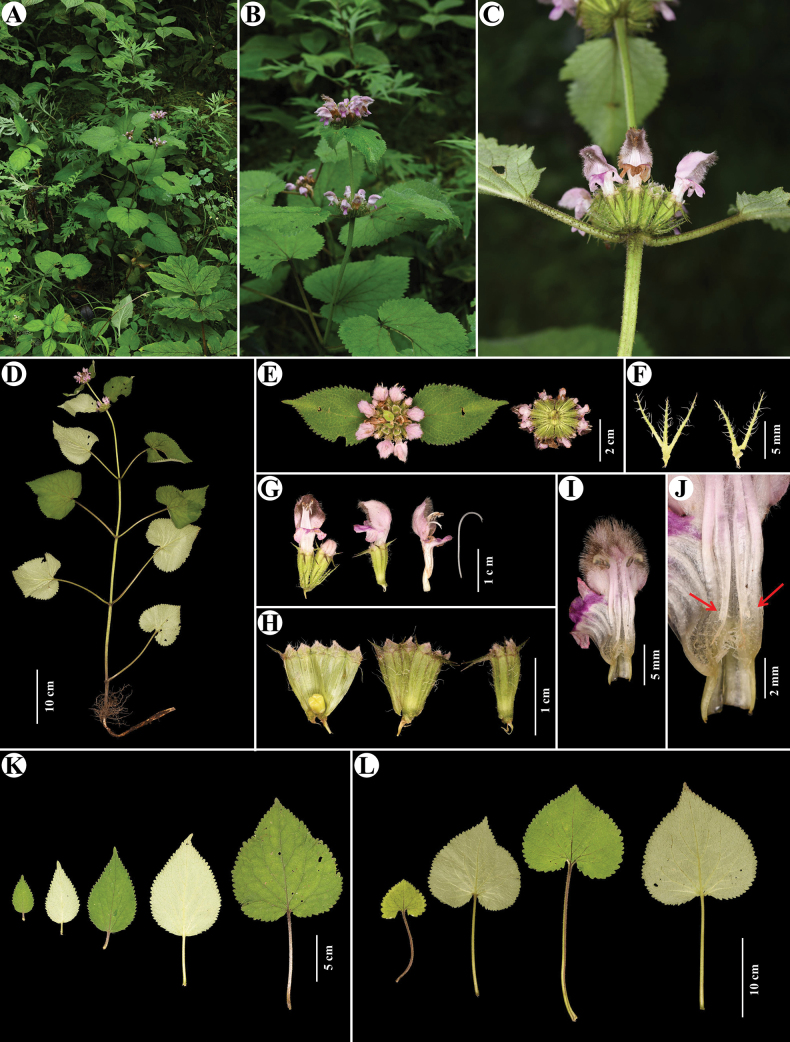
*Phlomoideslongidentata* Pendry **A** habitat **B, D** plant **C, E** verticillaster **F** bracts **G** flower **H** dissected calyces **I** dissected flower **J** appendages at base of posterior filaments (arrow) **K** floral leaves **L** stem leaves (Photographed by Yue Zhao).

#### Phenology.

Flowering from July to September, fruiting from October to December.

#### Distribution and habitat.

China, Bhutan and Nepal; new record for China found in Xizang (see below). It grows in forests and forest margins at altitudes between 2000 and 3800 m (Fig. 3).

#### Chinese name (assigned here).

cháng cì cǎo cāo sū (长刺草糙苏)

#### Additional specimens examined.

China. Xizang Province: • Rikaze City, Dingri County, Rongxia Town, 28°1′12.7″N, 86°15′52.1″E, alt. 2888 m, 29 Aug 2023, *Y. Zhao, R.Z. Bai, Q. Tian & M.L. Qian XCL2670* (KUN 1614352!, KUN 1614353!) • Rikaze City, Dingri County, Rongxia Town, 28°1′12.7″N, 86°15′52.1″E, alt. 2888 m, 11 Sep 2019, *Y.P. Chen, Y. Zhao & B.Y. Zhang EM1123* (KUN 1614351!) • Rikaze City, Dingri County, Rongxia Town, alt. 3200 m, 2 Aug 1959, *Anonymous 752* (PE 00832482!).

### ﻿Key to species of *Phlomoides* from subclade IIa

**Table:** 

1	Upper corolla lip with trichomes transparent to white	**2**
–	Upper corolla lip with trichomes brown to black	**3**
2	Stellate trichomes absent on leaves and bracts, plant often taller than 1 m	***P.henryi***
–	Stellate trichomes present on leaves and bracts, plant often shorter than 10 cm	***P.rotata***
3	Bracts and calyces with brown to black trichomes	**4**
–	Bracts and calyces with white trichomes	**5**
4	Bracts with black simple trichomes, stellate trichomes absent	***P.tibetica***
–	Bracts with brown simple and stellate trichomes	***P.milingensis***
5	Calyx teeth obviously emarginate	**6**
–	Calyx teeth subtruncate or slightly emarginate	**9**
6	Calyx with two longer and three shorter apical teeth	***P.longidentata***
–	Calyx with five equal teeth	**7**
7	Plant less than 0.5 m tall, basal leaves present; upper floral leaves with petiole ca. 2–5 mm long	***P.nana***
–	Plant often taller than 1 m, basal leaves absent; upper floral leaves lack petiole	**8**
8	Corolla purple	***P.nyalamensis***
–	Corolla white	***P.macrophylla***
9	Corolla less than l.5 cm long	***P.breviflora***
–	Corolla more than 2 cm long	***P.bomiensis***

## Supplementary Material

XML Treatment for Phlomoides bomiensis

XML Treatment for Phlomoides longidentata

## ﻿Additional information

### Author ORCIDs

Yue Zhao https://orcid.org/0000-0001-6850-5070

Ya-Ping Chen https://orcid.org/0000-0002-7502-1848

Alexander P. Sukhorukov https://orcid.org/0000-0003-2220-826X

Chun-Lei Xiang https://orcid.org/0000-0001-8775-6967

### Data availability

All of the data that support the findings of this study are available in the main text or Supplementary Information.

## Supplementary materials

Supplementary material 1 List of *Phlomoides* taxa sampled with information related to taxonomy, voucher information, and GenBank accession numbers Data type xlsx Explanation note GenBank accession numbers of the newly generated sequences are marked in bold, other sequences are taken from GenBank (– indicates missing information). https://binary.pensoft.net/file/1121815This dataset is made available under the Open Database License (http://opendatacommons.org/licenses/odbl/1.0/). The Open Database License (ODbL) is a license agreement intended to allow users to freely share, modify, and use this Dataset while maintaining this same freedom for others, provided that the original source and author(s) are credited.Yue Zhao, Ya-Ping Chen, Rui-Zhu Bai, Colin A. Pendry, Alexander P. Sukhorukov, Chun-Lei Xiang
